# Morphological patterns of circumpulpal dentin affected by radiation-related caries

**DOI:** 10.4317/jced.56584

**Published:** 2020-05-01

**Authors:** Isabel-Schausltz-Pereira Faustino, Natalia-Rangel Palmier, Patrícia-Maria Fernandes, Ana-Carolina-Prado Ribeiro, Thais-Bianca Brandão, Alan-Roger Santos-Silva, Pablo-Agustin Vargas, Marcio-Ajudarte Lopes Lopes

**Affiliations:** 1 DDS, MsC, PhD. Department of Oral Diagnosis, Piracicaba Dental School, University of Campinas, Piracicaba, Brazil; 2 DDS, MsC. Department of Oral Diagnosis, Piracicaba Dental School, University of Campinas, Piracicaba, Brazil; 3 DDS, MsC, PhD. Dental Oncology Service, Instituto do Câncer do Estado de São Paulo [ICESP], Faculdade de Medicina da Universidade de São Paulo, São Paulo, Brazil

## Abstract

**Background:**

The aim of this work was to evaluate the microscopic characteristics through polarized light microscopy, scanning electron microscopy and the mineral content of circumpulpal dentin of irradiated (IT) and non-irradiated teeth (NIT), with deep caries that reached the root canal.

**Material and Methods:**

A total of 25 IT were analyzed macroscopically, and radiographed. 5 NIT were used as controls. Two 100-μm-thick sections, per specimen, were evaluated in a polarized light microscopy and a scanning electron microscope. Demographics and clinical data were collected.

**Results:**

The results did not demonstrate distinct morphology of the IT compared with NIT. Mineral content values by weight percentage of Ca and P were also similar, corresponding to 66.65% and 33.21% in the IT and 66.60% and 33.29% in the NIT. The Ca/P ratio did not show statistical differences between groups being respectively 2.74 and 2.72, in the IT and NIT (*p*> 0.05).

**Conclusions:**

Radiotherapy does not change morphology and mineral content of circumpulpal dentin in IT.

** Key words:**Root canal, radiation-related caries, polarization microscopy, scanning electron microscopy, radiotherapy, oral neoplasms, dentin.

## Introduction

Radiation-related caries (RRC) is a challenging problem as it can lead to rapid coronary destruction and tooth loss in a short period of time (1). Much is said about whether RRCs are caused by a direct or indirect effect of radiotherapy on the dental structure, with current evidence pointing that indirect effect is particularly more important (1,2). An important additional factor is that RRCs may have a deceptively discreet clinical appearance, while in reality it demonstrates demineralized lesions with greater amplitudes when observed in microtomographies (3).

The management of RRC is challenging, since even when the teeth are amenable to restoration; there is a possibility of secondary caries development or detachment of the restorative material (4). On the other hand, tooth extraction should be avoided due to the risk of developing osteoradionecrosis (5). In this context, endodontic treatment is an option. Some studies, particularly case series, have been published in the English-language literature about endodontic treatment in irradiated teeth, evaluating microhardness and flexural strength of dentin and apical seal (6-13).

However, it has been observed clinically, difficulties in removing softened dentin tissue in the canal of irradiated teeth. There are also reports of reduction in the peak of phosphate according to deepening in irradiated dentin (14). Some reports have showed success and other failures of endodontic treatment related to radiation therapy (9,10).

Therefore, the objective of this study was to evaluate whether the morphology of deep RRC affecting circumpulpal dentin, (i.e, the layer of dentin around the outer pulpal wall) and the mineral content of irradiated teeth (IT) and non-irradiated teeth (NIT) to contribute to the better understanding of caries development in patients who underwent head and neck radiotherapy (HNRT).

## Material and Methods

-Sample selection

Twenty-five teeth from patients who received HNRT for squamous cell carcinoma at the State Cancer Institute of São Paulo (ICESP, São Paulo, SP, Brazil) were selected. In addition, 5 NIT of patients with head and neck cancer who were extracted during the oral pre-radiotherapy adequacy. All the teeth were extracted according to the individual dental needs of each patient, regardless of whether or not the research was carried out. In addition, all teeth had cavities that visually could affect the dental pulp. This characteristic was confirmed by digital periapical radiography (Sirona Dental Systems® GmbH, 20 Bensheim, Germany, with exposure time of 0.1s, and focus-film distance of 15cm). The ethical committee from *Pi*racicaba Dental School – São Paulo, Brazil reviewed and approved the present study.

Exclusion criteria included teeth with endodontic treatment and superficial caries.

-Clinical-demographic profile of patients

The irradiated teeth were from 13 patients who received a minimum dose of 50 Gy, with a mean of 66.30 Gy, all were treated with 3D modality of radiotherapy (Sinerg Plataform Linear accelerator, 6mV, Elekta AB, Stockholm, Sweden). The sample consisted mainly of males (84.61%), ranging in age from 49 to 77 years, with a mean of 57.53 years. The most prevalent primary tumor site was oropharynx (7 cases, 53.84%) followed by larynx (3 cases, 23.07%) and oral cavity (3 cases 23.07%), and all of them were in advanced clinical staging as follow: stage IVA (8 cases - 61.53%), stage IVB (3 cases - 23.07%) and stage III (2 patients - 15.38%).

The non-irradiated teeth were extracted from 5 patients during comprehensive dental treatment prior to HNRT, whose sample was composed only of male patients (100%), ranging in age from 41 to 81 years, with a mean of 54 years. The most prevalent primary tumor site was oropharynx (3 cases, 60%) followed by larynx (2 cases, 40%). Two cases (40%) were diagnosed in the IVA stage, followed by 1 case (20%) each in the stage II, III and IVB. Complete information on patients’ clinical and demographic profile is listed in Table 1.

**Table 1:**
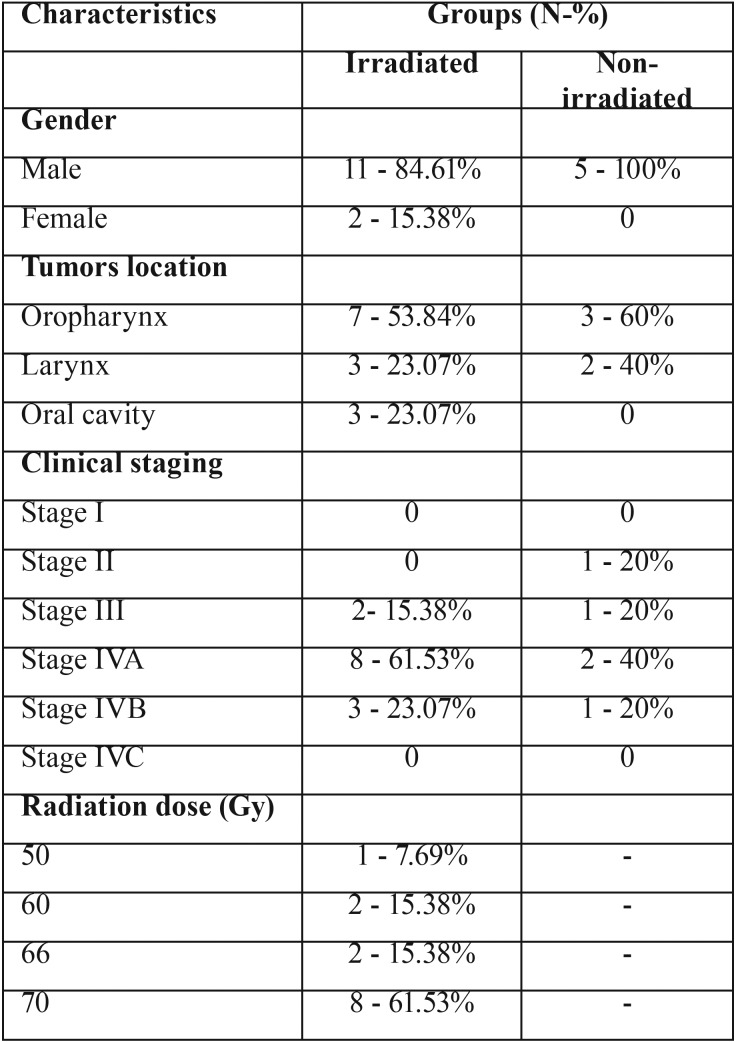
Clinical and demographic profile of samples.

-Preparation of the samples

The teeth were cleaned with periodontal curettes to remove soft tissue debris and later, each tooth was longitudinally sectioned in the vertical plane with the aid of a diamond disc of high concentration (Extec, Enfield, CT, USA) in precision cutter (Isomet 1000- Buehler Ltda., Lake Bluff, IL, USA). Two cuts of approximately 1mm were made for analysis in polarized light microscopy (POLMI) and scanning electron microscopy (SEM). The surface of the slices was polished with silicon carbide sieves in the granulations 600; 1,200; 2,000 and 4,000 for planning and obtaining a thickness of approximately 100μm. The thickness standardization was controlled using a digital caliper (Standard Gage, Hexagon Metrology, Stockholm, Sweden).

-Polarized light microscopy (POLMI)

For visualization of specimens under a POLMI (DM 5.000, Leica, Wetzlar, Germany) a cut of approximately 100μm of each tooth was prepared, as described in the preparation of the sample, immersed in deionized water and placed on a glass slide and under it, a cover slip.

In a 50x magnification, the circumpulpal dentin was evaluated by scanning in the entire length of the canal and analyzed from the expected birefringence for mineralized and demineralized dentin and the presence or not of reparative dentin. All the generated images were captured with a digital camera coupled to the polarized light microscope and analyzed using the Leica Qwin image (Leica Application Suite Cambridge, UK) capture program.

-Scanning electron microscopy (SEM)

A cut of approximately 100μm of each sample, prepared as described in the sample preparation, was used for dentin surface evaluation. The prepared samples were fixed with double carbon tape (Electron Microscopy Sciences, Washington 19034 - USA) in acrylic stubs for storage at 40 ° C for drying for 12 hours in a hothouse (Model 315 SE – Fanem, SP, Brazil). After drying, the samples were covered with carbon using the evaporator equipment (Balzers SCD 050 sputter coater, Balzers Union Aktiengesellschaft, Furstentum Liechtenstein, FL-9496 - Germany), with a current of 45mA, for 160 seconds.

The specimens were then analyzed by SEM (JSM – 5600LV – JEOL LTD., Tokyo, Japan) with the use of backscattered electrons at a voltage acceleration of 15KV, work distance of 20mm and spotsize of 43nm with a minimum increase of 35 times. The observation by SEM consisted of analysis of the circumpulpal dentin by scanning in the entire length of the canal from the difference of expected shades of gray for mineralized and demineralized dentin and the presence or not of reparative dentin.

-Microanalysis based on energy dispersion spectroscopy

The mineral content analysis was performed with the aid of the Vantage microanalysis system (NORAN Instruments, Middleton, WI., USA) in association with the scanning electron microscope, where the generation of the quantification of the elements Calcium (Ca) and Phosphorus (P). Quantification was obtained as percentage of each element in two different random and contralateral areas (spot size with average of 16.500 µm²) of each site of circumpulpal dentin evaluated in the irradiated and non-irradiated specimens. Then, two random quantifications were performed in the coronal circumumpulpar dentin, two random quantifications in medium region and two random quantifications in apical region in the two groups of specimens. The counting time in each area was 100 seconds, with PHA deadtime ranging between 20 and 25%.

Values of Ca and *P* were obtained and the average values were calculated per area and sample. In addition, the Ca/P ratio for each area studied in each specimen and the general averages of Ca, *P* and Ca/P ratio by area studied were determined.

-Statistical analysis

Paired samples t test was used attempting to relate the mean values of percentage by weight Ca and *P* and Ca/P ratio among the thirds within the irradiated and non-irradiated groups. Independent samples t test was used to verify the correlation of mean values of the percentage by weight of Ca and *P* and Ca / *P* ratio by the equivalent thirds between the irradiated and non-irradiated groups. Statistical analysis was performed using the program MedCalc Statistical Software version 14.8.1 (MedCalc Software bvba, Ostend, Belgium; http://www.medcalc.org; 2014). For both tests, a significant value of *p*≤0.05 was considered.

## Results

-Polarized light microscopy

**Figure 1:**
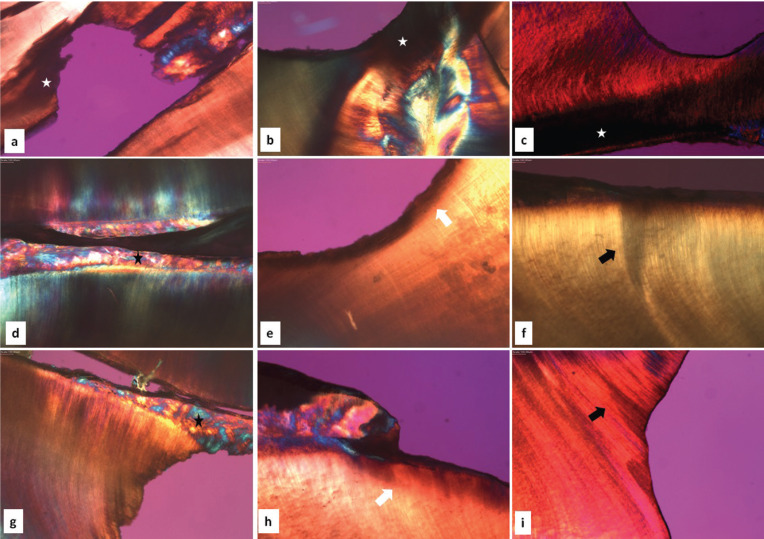
Polarized light microscopy. Pattern of birefringence loss in demineralized areas, showing dark areas and loss of tissue (white star) in irradiated (A, B) and non-irradiated (C) teeth (5x objective). Dentin reactions such as reparative dentin (black star), dentin sclerosis (white arrow) and dead tracts (black arrow) in irradiated (D, E, F) and non-irradiated (G, H, I) teeth (5x objective).

In the morphological analysis performed by POLMI, different patterns of birefringence between healthy dentin and carious dentin were observed in the group of IT and NIT, with black and opaque areas being related to the areas of demineralized dentin. Demineralization patterns in triangular and “half-moon” shapes were observed in both groups. There were no visual differences between the IT and NIT groups (Fig. 1 A-C).

In addition, the morphology of dentinal reactions to aggressions, such as reparative dentin, sclerotic dentin and dead tracts, were also similar in both groups: irradiated and non-irradiated (Fig. 1 D-I).

-Scanning Electron Microscopy

**Figure 2:**
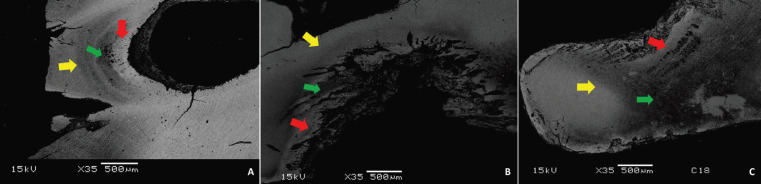
Scanning electron microscopy. Patterns of caries layers observed by changes in gray tones between mineralized and demineralized areas in irradiated (A, B) and non-irradiated (C) teeth. Surface layer (yellow arrow), intermediate layer of demineralization (green arrow) and sclerotic layer (red arrow).

In the morphological analysis performed by the SEM it was observed different patterns between healthy dentin and carious dentin in the groups of IT and NIT. The areas in dark pattern being related to the areas of demineralized dentin and white areas related to the mineralized areas, but without visual differences between the irradiated and non-irradiated groups. Caries lesions showed a black color gradient in their most demineralized portion, from gray to white in mineralized areas. The different layers of caries could be observed in both irradiated and non-irradiated groups such as surface or external layer (area with increased mineral density), intermediate or deep layer (area with decrease of mineral density) and sclerotic or transparent layer (area with increased mineral density). In areas of diffuse mineral density loss, caries zones were not well demarcated in the IT and NIT. The intermediate layers, characterized by demineralized tissue, showed dentin tubules with increased diameter (Fig. 2).

**Figure 3:**
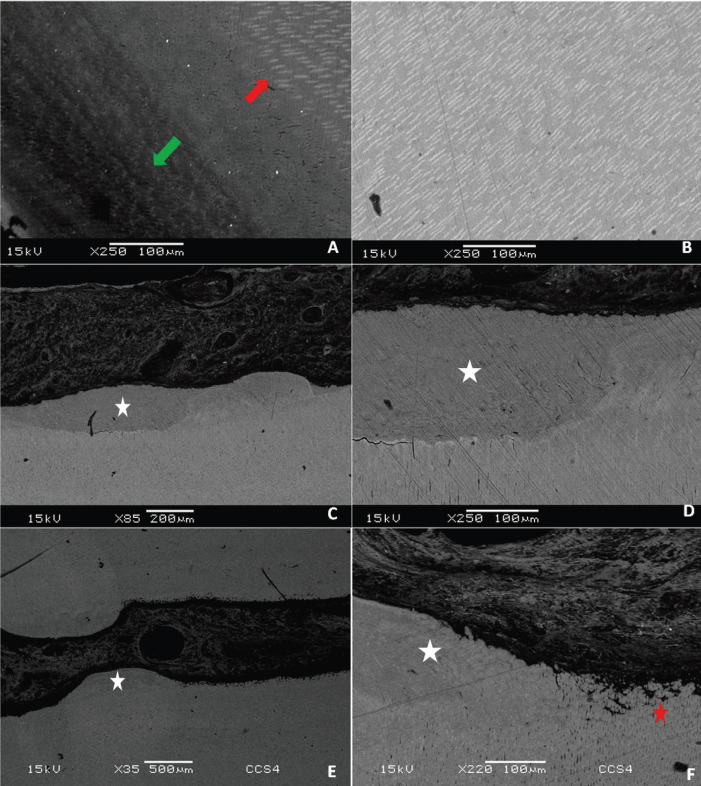
Scanning electron microscopy. Dentin reactions such as sclerosis (A,B) and reparative dentin (C,D,E,F). Interface zone between demineralized intermediate layer (green arrow) and deep layer of dentin sclerosis (red arrow) (A). Large amplification in the area of dentin sclerosis showing peritubular dentin deposition (B). Tooth irradiated with reparative dentin (white star) (C) and large amplification of the same reaction area (D). Non-irradiated tooth with reparative dentin (white star) (E) and amplification of interface area between termination of reparative dentin and pre-dentin (red star) (F).

As with dentin sclerosis, reparative dentin was observed in IT and NIT with similar microscopic pattern, demonstrated as dentin tissue proliferations into the root canal with or without dentinal tubules (Fig. 3).

-Microanalysis based on energy dispersion spectroscopy

In the group of IT, the overall average percentage by weight of Ca and P, obtained from the averages of each area, was 66.65% and 33.21%, respectively. Statistical analysis comparing the percentage of Ca and *P* weight among the thirds demonstrated statistical difference between coronal versus middle (*p*=0.0350 / *p*=0.001, respectively) and coronal versus apical (*p*=0.0221 / *p*= 0.0027, respectively).

In the group of NIT, the overall average percentage by weight of Ca and P, obtained from the averages of each area, was 66.60% and 33.39%, respectively. Statistical analysis comparing the percentage of Ca and *P* weight among the thirds demonstrated statistical difference between coronal versus apical (*p*=0.0440 / *p*=0.0440) and middle versus apical (*p*=0.0487) only for P.

Although the mean values of Ca and *P* were lower in the IT than in the non-NIT, there was no statistically significant difference (*p*> 0.05).

In the IT the overall mean Ca/P ratio, obtained from the averages of each area, was 2.74 and standard deviation 0.04. Statistical analysis of Ca/P ratio among the areas demonstrated: coronal versus middle (*p*=0.0178), coronal versus apical (*p*=0.0025), middle versus apical (*p*=0.0680).

In the group of NIT, the overall mean Ca/P ratio, obtained from the averages of each area, was 2.72 and standard deviation 0.06. Statistical analysis of Ca/P ratio among the areas demonstrated: coronal versus middle (*p*=0.3262), coronal versus apical (*p*=0.0291), middle versus apical (*p*=0.0256). There was no statistical difference between the mean values between the IT and NIT (*p*> 0.05).

The summary of the information obtained by the microanalysis based on the energy dispersion spectroscopy is summarized in Table 2 and  Table 3.

**Table 2:**
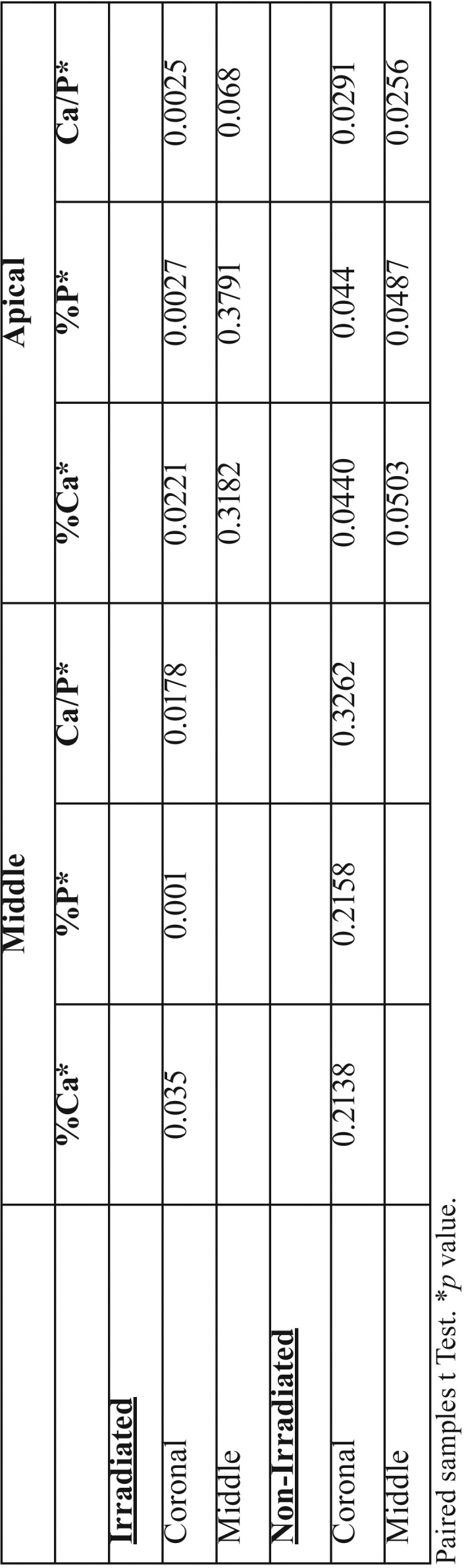
*p* value from statistical analysis of mean values of percentage by weight Ca and P and Ca / P ratio among the thirds within the irradiated and irradiated groups.

**Table 3:**
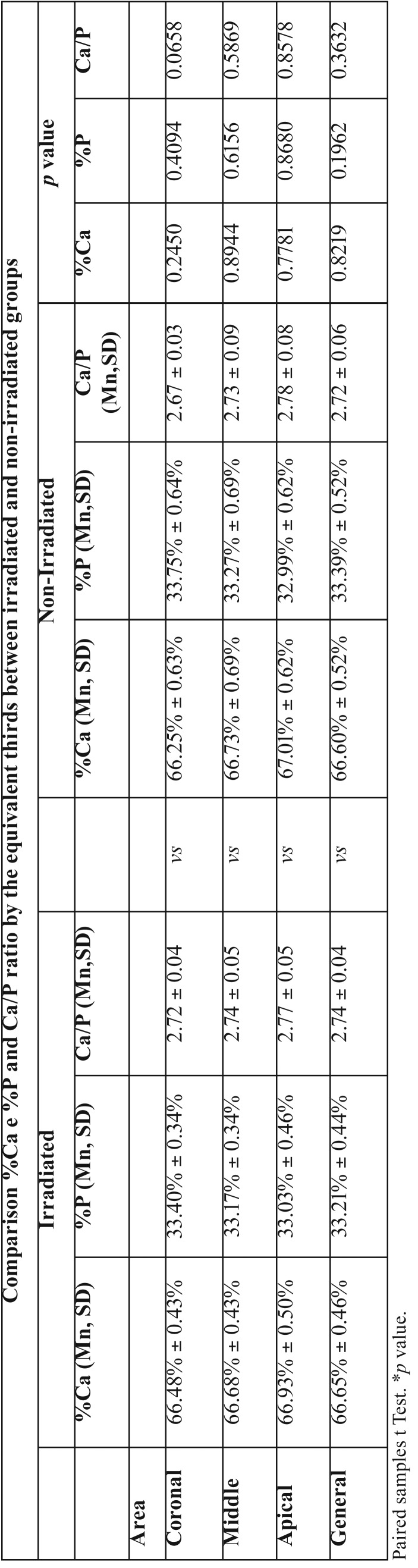
Comparison of the mean values of the percentage by weight of Ca and P and Ca / P ratio by the equivalent thirds between the irradiated and non-irradiated groups.

## Discussion

POLMI is a sensitive technique that can show changes in tooth structure through direct visualization of birefringence, where the healthy tooth is bright in polarization, while areas with mineral loss appear opaque and dark when compared to each other. In the analysis of caries lesions by polarized light microscopy, with samples prepared through wear, there are usually some areas related to the progression of caries lesion in dentin, characterized by the amount of mineralization. Areas of softened dentin on the surface of the caries cavity, demineralized dentin, dentin sclerosis, translucent area, dentinal dead tracts and secondary dentin may be observed, most often exhibiting a triangular and “half-moon” shape (15,16). The described dentin reactions were observed in our sample with similar visual appearance between the IT and NIT, demonstrating that the pattern of dental response to caries of IT is not differentiated from NIT. In deep lesions, caries areas are not well demarcated due to substantial tissue loss. However, there was no difference between groups. In addition, our sample exhibited the same pattern of shape and lesion layers observed by SEM in the IT and NIT. The deep lesions of the IT that reached the root canal demonstrated a demineralization pattern similar to that found in the NIT, signaling that the deep lesions of caries on irradiated teeth did not have a different morphological pattern.

The deleterious action of radiation under the ability of the dentin to react to external stimuli has been contradictory, with studies indicating possible decrease of the reactional capacity and others indicating capacity similar to NIT (1). Our results demonstrate the presence of the dentin reactions and dead tracts in the irradiated and non-irradiated groups, showed that the dentin retains its reactive activity, reinforcing the idea that odontoblast function is not compromised after tooth exposure to radiation.

Patients who will receive HNRT as planning therapy for head and neck cancers should undergo a specialized dental evaluation in order to identify teeth with RRC development potential, thereby minimizing the likelihood of post-radiotherapy exodontia and consequent increase in the susceptibility to the development of osteoradionecrosis. Since the RRC arises, the restorative therapies are described with low success rate due to the detachment of the restoration or secondary caries, perpetuating the probability of infection in periapex (4). However, many patients are not evaluated pre-radiotherapy and those who are evaluated, however, may develop RRC and consequent sequelae of infection. Many teeth are then referred to endodontic therapy to avert tooth extraction in order to avoid possible negative repercussions of the surgical procedure.

Our results demonstrate that the lesions of deep caries present in the same form in IT and NIT through morphological analysis. However, clinical experience have demonstrated the presence of softened dentin in the root canal of irradiated teeth, which impairs the canal sealing process, directly influencing the success of endodontic treatment. Softened dentin is characterized by demineralization, which is influenced by the mineral loss of Ca and *P* as well as reduction of the Ca/P ratio in relation to the healthy dentin (19). In this context, we analyzed the mineral content of Ca and P of the dentin that covers the entire root canal of teeth with deep caries of IT and NIT. The results showed there was a statistical difference in the percentage by weight of Ca and P and Ca/P ratio between the coronal, middle and apical thirds in both IT and NIT. This can be explained according to the progression of caries from the coronal third to the lower thirds. Although the mean values of the percentage by weight of Ca and P were lower in the IT than NIT, there was no statistically significant difference between groups. The Ca/P ratio was lower in the general mean in NIT than in the IT, but in the same way as the percentage by weight of Ca and P, there was no statistically significant difference between the groups. Although the data of the literature are with very close values between demineralized dentin, pre-dentin and healthy dentin (18,19), our results corroborate with the variation of the Ca/P ratio values for dentin caries lesions described in the literature (18). In addition, it has been described that human enamel submitted to different doses of radiation does not show alteration in calcium and phosphorus values (20). Recent study evaluating the mineral content of human root dentin in irradiated teeth found that after radiation there is a reduction in Ca and P values (21). However, the specimens used were irradiated *in vitro*, which does not reproduce the reality of soft tissue protection and consequent total dose of radiation in the tooth.

In general, dental caries is considered to be a multifactorial disease, characterized by tissue destruction from the action of bacterial acid by-products from the fermentation of dietary carbohydrates (22). For caries to progress or suffer reversal, an imbalance between remineralization and demineralization processes must occur. In order to avoid the establishment of caries, remineralization must occur and for it to occur, saliva is required, which restores the pH of the biofilm, acting as a buffer effect. In addition, saliva returns calcium and phosphate to the tooth that has undergone the demineralization process (23,24). Thus, some risk factors for the onset of caries can be recognized, such as inadequate composition and salivary flow, increased number of cariogenic bacteria, insufficient exposure to fluoride, gingival recession, immunological competence, lack of hygiene and genetic component (22,25). Based on current results, the radiation probably does not have a direct effect on the dental structure capable of altering the morphological patterns of demineralization. However, the known deleterious role of radiation on the salivary glands, the immunological status of patients with head and neck cancer, dietary alterations and morbidities related to cancer treatment, seems to be more related to the emergence of caries in IT than the direct damage to the dental structure, conFiguring an indirect action of the radiation. This corroborates with the indication that the presence of the RRC is not defined by the presence of the tooth in the field of irradiation, but the presence of the main salivary glands as a possible main factor for the emergence of RRC (26).

In conclusion, deep caries alterations, dentinal reactions and mineral content in circumpulpal dentin of IT were similar to NIT. Therefore, it was not possible to attribute that the radiation causes direct effect on the circumpulpal dentin.
